# Bacilluria without Tuberculosis of the Urinary Passages

**Published:** 1914-08

**Authors:** 


**Bacilluria Without Tuberculosis of the Urinary Passages.** Rist and Kindberg, Gaz. des Prat., April 1, 1914, report a case of a woman who had several crises of asystole of aortic origin and who died of intercurrent erysipelas. Tubercle bacilli had been absent in the expectoration, even after making cultures but the antigen reaction was present and the urine twice produced tuberculosis in guinea pigs. One minute histologic examination, post mortem and attempted inoculation of guinea pigs with various organs, no tuberculosis could be found except an old cicatrized focus in the left pulmonary apex, from which inoculation was successful.

